# Variation Between Hospitals in Outcomes and Costs of IBD Care: Results From the IBD Value Study

**DOI:** 10.1093/ibd/izae095

**Published:** 2024-04-26

**Authors:** Reinier C A van Linschoten, C Janneke van der Woude, Elyke Visser, Nikki van Leeuwen, Alexander G L Bodelier, Claire Fitzpatrick, Vincent de Jonge, Hestia Vermeulen, K Evelyne Verweij, Sanne van der Wiel, Daan Nieboer, Erwin Birnie, Daniëlle van der Horst, Jan A Hazelzet, Desirée van Noord, Rachel L West

**Affiliations:** Department of Gastroenterology and Hepatology, Franciscus Gasthuis and Vlietland, Rotterdam, the Netherlands; Department of Gastroenterology and Hepatology, Erasmus MC, Rotterdam, the Netherlands; Department of Gastroenterology and Hepatology, Erasmus MC, Rotterdam, the Netherlands; Department of Gastroenterology and Hepatology, Franciscus Gasthuis and Vlietland, Rotterdam, the Netherlands; Department of Gastroenterology and Hepatology, Erasmus MC, Rotterdam, the Netherlands; Department of Public Health, Erasmus University Medical Centre, Rotterdam, the Netherlands; Department of Gastroenterology and Hepatology, Amphia Hospital, Breda, the Netherlands; Department of Gastroenterology and Hepatology, IJsselland Hospital, Capelle aan de IJssel, the Netherlands; Department of Gastroenterology and Hepatology, Albert Schweitzer Hospital, Dordrecht, the Netherlands; Department of Gastroenterology and Hepatology, Ikazia Hospital, Rotterdam, the Netherlands; Department of Gastroenterology and Hepatology, Maasstad Hospital, Rotterdam, the Netherlands; Department of Gastroenterology and Hepatology, Reinier de Graaf Gasthuis, Delft, the Netherlands; Department of Public Health, Erasmus University Medical Centre, Rotterdam, the Netherlands; Department of Statistics and Education, Franciscus Gasthuis and Vlietland, Rotterdam, the Netherlands; Department of Genetics, University Medical Centre Groningen, University of Groningen, Groningen, the Netherlands; Crohn & Colitis NL, Woerden, the Netherlands; Department of Public Health, Erasmus University Medical Centre, Rotterdam, the Netherlands; Department of Gastroenterology and Hepatology, Franciscus Gasthuis and Vlietland, Rotterdam, the Netherlands; Department of Gastroenterology and Hepatology, Franciscus Gasthuis and Vlietland, Rotterdam, the Netherlands

**Keywords:** inflammatory bowel disease, value-based healthcare, variation, advanced therapy, quality improvement

## Abstract

**Background:**

Data on variation in outcomes and costs of the treatment of inflammatory bowel disease (IBD) can be used to identify areas for cost and quality improvement. It can also help healthcare providers learn from each other and strive for equity in care. We aimed to assess the variation in outcomes and costs of IBD care between hospitals.

**Methods:**

We conducted a 12-month cohort study in 8 hospitals in the Netherlands. Patients with IBD who were treated with biologics and new small molecules were included. The percentage of variation in outcomes (following the International Consortium for Health Outcomes Measurement standard set) and costs attributable to the treating hospital were analyzed with intraclass correlation coefficients (ICCs) from case mix–adjusted (generalized) linear mixed models.

**Results:**

We included 1010 patients (median age 45 years, 55% female). Clinicians reported high remission rates (83%), while patient-reported rates were lower (40%). During the 12-month follow-up, 5.2% of patients used prednisolone for more than 3 months. Hospital costs (outpatient, inpatient, and medication costs) were substantial (median: €8323 per 6 months), mainly attributed to advanced therapies (€6611). Most of the variation in outcomes and costs among patients could not be attributed to the treating hospitals, with ICCs typically between 0% and 2%. Instead, patient-level characteristics, often with ICCs above 50%, accounted for these variations.

**Conclusions:**

Variation in outcomes and costs cannot be used to differentiate between hospitals for quality of care. Future quality improvement initiatives should look at differences in structure and process measures of care and implement patient-level interventions to improve quality of IBD care.

**Trial Registration Number:**

NL8276

**KEY MESSAGES**

**What is already known?**

Unwarranted treatment variation is a major barrier for providing (cost-)effective care for patients with inflammatory bowel disease (IBD), and comparing data on outcomes and costs can be used to improve the quality of care.

**What is new here?**

We show that relying only on outcome measurements is insufficient for informing quality improvement initiatives in IBD care within hospitals.

**How can this study help patient care?**

Our study shows deficits in quality of IBD care, and we recommend looking at differences in structure and process measures of care and implementing patient-level interventions to improve the quality of IBD care.

## Introduction

Inflammatory bowel disease (IBD) is a complex disease and has considerable impact on the quality of life of patients.^1,2^ The introduction of advanced therapies, biologics and new small molecules, not only has revolutionized the treatment of IBD, but also contributes to the rapidly rising healthcare costs of IBD.^3,4^ As the prevalence of IBD is predicted to double in the Western world within the next two decades, healthcare systems are under increasing financial pressure.^5,6^ Initiatives to improve (cost-)effectiveness of care for patients with IBD are urgently needed to keep care affordable, accessible, and of high quality.^7^

A major barrier for providing (cost-)effective care for patients with IBD is unwarranted treatment variation.^8-10^ Prior studies have shown substantial variation in structure and process measures related to the treatment of IBD.^11-17^ These include variation in the presence of dedicated IBD units and access to advanced therapies across European hospitals.^11^ Variation has also been found in the use of therapeutic drug monitoring, medication prescription, and dysplasia management between providers.^12-16^ Unwarranted variation in care leads to suboptimal outcomes and unnecessary healthcare use.^18^ This is illustrated by a prior study on outcomes of IBD care that showed better results for patients with Crohn’s disease treated by gastroenterologists in an academic setting compared with nonacademic providers in the United States, although this was not observed in patients with ulcerative colitis.^17^ Addressing and reducing variation in treatment could improve outcomes and reduce costs for patients with IBD.^18^

One strategy to reduce variation and improve (cost-)effectiveness of IBD care is value-based healthcare. Value-based healthcare aims to improve technical value, defined as the health outcomes achieved divided by the cost of treatment over the entire care cycle. Benchmarking is a promising tool to improve value. It involves the comparison of outcomes and costs of treatment among different hospitals.^19-21^ Hospitals can use these data on outcomes, costs, and variation to identify areas for improvement, learn from each other, and reduce unwarranted variation.

To measure outcomes of IBD care that matter to patients and compare these between providers, the International Consortium for Health Outcomes Measurement (ICHOM) has developed a standard set of IBD outcomes that includes both clinician- and patient-reported outcome measures and a case mix adjustment set.^22^ This set was developed with patients and specialists to compare meaningful outcomes of IBD treatment between hospitals. A previous study demonstrated the feasibility of using the patient-reported outcomes from this standard set to identify shortcomings in IBD care in a group of National Health Service (NHS) hospitals. Using the standard set, they found that a much higher proportion of patients (8%) were on long-term steroids than previously expected, highlighting the need for interventions to reduce steroid dependency.

However, no studies have used the consensus-based ICHOM standard set to robustly assess variation in outcomes and costs of IBD care. We conducted the IBD Value study to assess variation in outcomes and costs of IBD treatment for patients on advanced therapies between different treating hospitals to identify deficits in care and create a benchmark for future quality improvement programs.^23^

## Methods

### Study Design

The IBD Value study was a multicenter longitudinal nonrandomized parallel cluster trial conducted to assess variation in outcomes and costs of IBD care for patients on advanced therapies between different treating hospitals to improve quality of IBD care (see also the published study protocol).^23^ The variation in outcomes and costs of IBD treatment was assessed in a 12-month period from December 2020 to December 2021 in 8 hospitals in the Southwest region of the Netherlands. These hospitals have collaborated in the IBD BeterKeten since 2016 to improve quality of care. In our collaboration, discussions on IBD care showed variation in the treatment and follow-up of patients. This project served as an initial effort for a better understanding of differences between the hospitals and pave the way for moving toward more standardized care, ultimately aiming to enhance the quality of IBD care. The participating hospitals are diverse, encompassing 2 community, 5 large teaching, and 1 academic hospital(s). These hospitals cater to both urban and rural areas, serving patients from a broad spectrum of ethnic, socioeconomic, and educational backgrounds.^24^

### Patients

Patients were included in the study between November 2020 and December 2021. Patients could be included at any time point during the study period. Patients were eligible for the study if they had an IBD diagnosis for at least 3 months, were at least 18 years of age, and were being treated with an advanced therapy at any point during the study period. All patients were on advanced therapies when they were included. The patient population thus included those who were biologic naïve, starting induction treatment at inclusion in the study, as well as patients who had been on maintenance therapy with advanced therapies for an extended period. Advanced therapies included infliximab, adalimumab, golimumab, vedolizumab, ustekinumab, tofacitinib, filgotinib, and ozanimod. Patients that could not complete questionnaires due to insufficient knowledge of the Dutch language or who did not have Internet access were excluded.

### Outcomes

The ICHOM standard set was used to measure outcomes of IBD treatment, including IBD-attributable mortality, colorectal cancer, anemia, endoscopic/radiologic remission (no inflammation on endoscopy or radiology), biochemical remission (fecal calprotectin below 100 μg/g for Crohn’s disease and 250 μg/g for ulcerative colitis), clinical remission as reported by the healthcare provider, patient-reported remission according to the Manitoba IBD Index, accident and emergency (A&E) visits, hospital admissions, length of stay, long-term steroid use (cumulative use of 3 months per year), complications of IBD treatment, patient-reported disease control according to the IBD-Control questionnaire, fistulae symptoms, and body mass index.^25-28^ The ICHOM set was supplemented with generic health-related quality of life, costs, and patient experiences with care. These were general, physical, mental and social health according to the PROMIS-10 (10-item Patient-Reported Outcomes Measurement Information System Global Health) questionnaire; health-related quality of life and utility according to the EQ-5D-5L; and healthcare costs, productivity costs, patient costs, and experiences with care according to the patient experience monitor.^29-32^ Healthcare costs were split between hospital costs and primary care costs, with hospital costs consisting of admissions, surgery, day treatment, outpatient clinic visits, diagnostics, medication, and A&E visits for IBD-related specialties. See Supplementary Table 1 for a detailed list of outcomes, sources, and definitions.

### Case Mix

To account for case mix differences when comparing hospitals, we adjusted all analyses for outcomes and costs for the case mix variables as specified by ICHOM. These are age, sex, educational level as defined by UNESCO,^33^ smoking status, comorbidities as measured by the Self-Administered Comorbidity Questionnaire (SCQ),^34,35^ diagnosis, Montreal classification, extraintestinal manifestations, diagnosis of primary sclerosing cholangitis, and current or prior infection with hepatitis B, tuberculosis, and human immunodeficiency virus. See the Supplementary Methods and Supplementary Table 2 for source of the variables (patient-reported or chart review) and more details. Analyses for experiences with care were not adjusted for patient characteristics, as case mix variables should not influence whether patients are treated in a patient-centered manner.

### Timing and Procedures

Our study aimed to investigate outcome variations in real-world settings among different hospitals. To understand how differences in treatment and follow-up practices among hospitals could impact outcomes, we did not standardize patient monitoring. Instead, each healthcare provider continued treatment according to their local protocols and independently determined the monitoring frequency, procedures, and timing for escalating therapy for their patients. Case mix questionnaires were sent out at inclusion. Surveys for the patient-reported outcomes were sent out at months 6 and 12. Cost questionnaires were sent out every 3 months. The patient experience questionnaire was sent to each patient once during the 12-month period; this questionnaire was sent out after an outpatient clinic visit. Clinician-reported outcomes were retrieved from the electronic health records retrospectively and administrative data were linked to the study database using custom developed open-source software.^36^

### Statistical Methods

No formal sample size calculation was done, but we aimed to include as many patients as possible to improve generalizability and power to find variation in outcomes.

Patients were included in the analysis at the 6-month time point if they had been included in the first 6 months of the study and at the 12-month time point if they had been included in the first 12 months of the study, as they would otherwise not have received the surveys for the patient-reported outcome measures. Missing data for case mix variables was multiply imputed.^37,38^ Outcomes, costs, and experiences with care were analyzed with (generalized) linear mixed models. All remission, anemia, survey, and cost data were analyzed per 6-month period, while A&E visits, admissions, long-term corticosteroid use, and complications of IBD treatment were analyzed over the entire 12-month study period. All models contained a random intercept at the hospital level to account for clustering and to assess variation between hospitals. We considered patients who transferred between hospitals in the random effects structure. None of the patients received treatment at multiple hospitals simultaneously. Models for data that were analyzed per 6-month period also contained a random intercept at the patient level to account for correlation between observations of the same patient and a fixed effect to account for the period in which the data were measured. For more details on the imputation procedure, model specification, and checking of the model assumptions, see the Supplementary Methods and Supplementary Table 3.

The random effects were used to calculate an intraclass correlation coefficient (ICC). The ICC indicates how much of the variation in the outcome, after case mix correction, can be ascribed to unmeasured patient characteristics (ie, the patient-level random effect) and hospital characteristics (ie, the hospital-level random effect). If the ICC at the hospital level increases, a larger portion of the observed variation in outcomes and costs between patients is associated with the treating hospital, and higher variability between hospitals can be expected.^39^ The patient-level random effect aims to capture unmeasured patient characteristics (ie, patient-specific characteristics that differ between patients and influence the outcome but were not captured by the case mix variables). A sensitivity analysis on the dichotomized IBD-Control-8 score was performed, with a score of 13 and higher indicating good disease control, as distribution of the residuals showed heteroscedasticity when using a linear mixed model.

We also performed post hoc analyses, in which we assessed the effect estimates of the lowest- and highest-performing hospitals as compared with the mean performance to show the absolute variation in outcomes, costs, and experiences.^40^ Highest and lowest performers are defined as the hospitals that have the highest and lowest effect estimate for each outcome. However, we should emphasize that no causal statement on quality of care of each of these individual hospitals can be made on the basis of these analyses. Effect estimates are reported as odds ratios for binomial models, differences for linear models, and relative differences for all other models. A relative difference of 0.8 for costs would indicate that the costs in this hospital are on average 80% of the mean costs. We used 95% confidence intervals to assess whether the lowest or highest performers were significantly different from the mean performance. Performers were significantly different from the average if their 95% confidence interval did not contain the value of no difference. These values are 0 for linear models and 1 for odds ratios and relative differences.

### Patient and Public Involvement

The Dutch Crohn’s and Colitis patient association (Crohn & Colitis NL) was involved in the study design and helped test the questionnaires for readability and length before start of the study. They were also involved in interpretation of the data and writing of this report.

## Results

**Figure 1.**
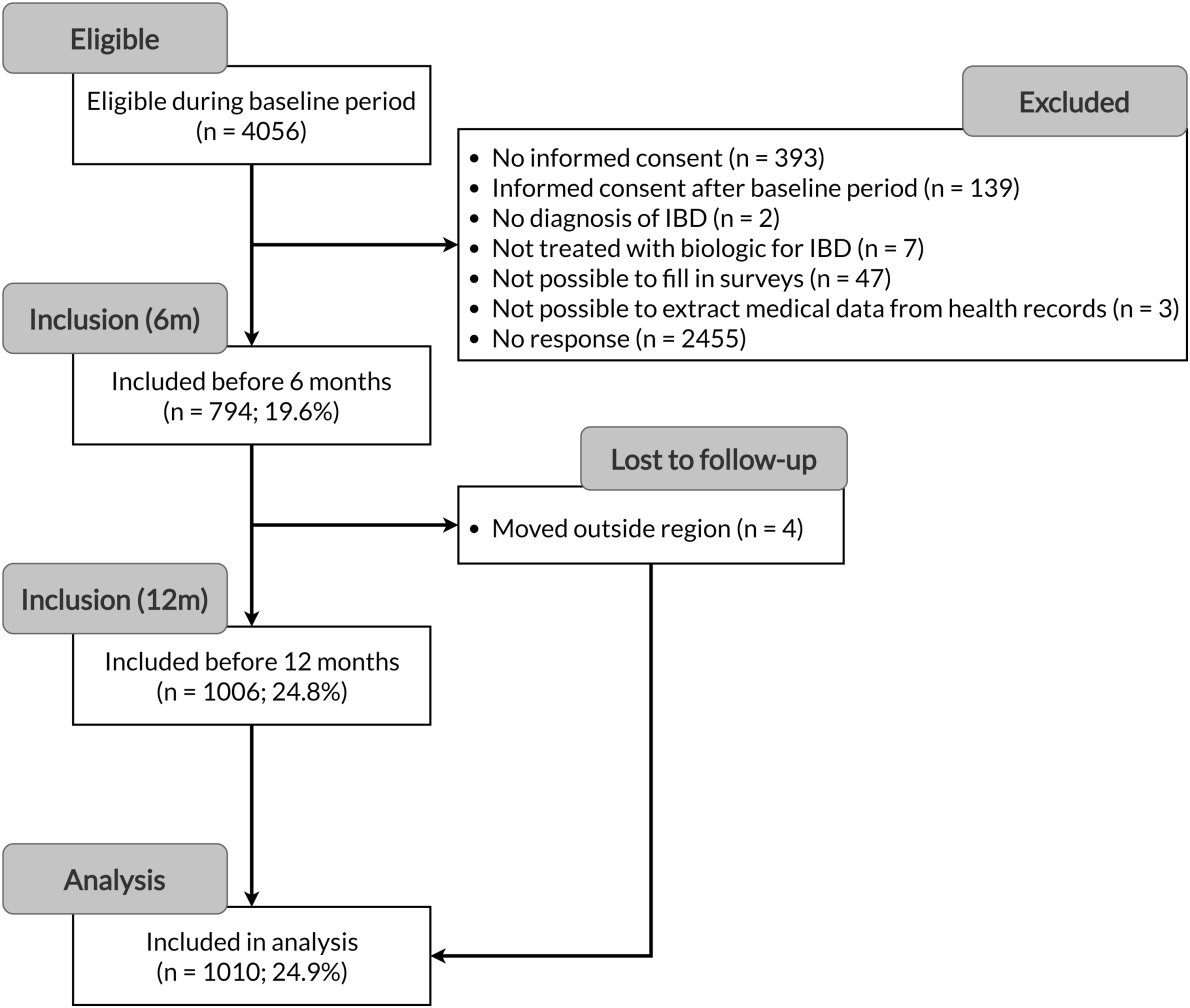
Flow chart of patient inclusion. IBD, inflammatory bowel disease; m, months.

Between November 2020 and December 2021, a total of 1010 patients were enrolled in the study; this is almost 25% of the entire eligible population (ie, adult IBD patients on advanced therapies) (Figure 1).

Baseline characteristics of patients are shown in Table 1; for baseline characteristics per hospital, see Supplementary Table 4. There were considerable differences in case mix variables between patient populations of the participating hospitals in terms of diagnosis, Montreal classification, extraintestinal manifestations, lifestyle factors, and treatment factors. For example, the percentage of female patients per hospital varied between 47% and 66%, the percentage of patients with Crohn’s disease varied between 54% and 75%, and the percentage of patients with primary sclerosing cholangitis varied between 0% and 7.2%. A relatively high level of education was seen in the overall patient population. Approximately 1 in 6 patients were active smokers. The occurrence of previous infectious diseases was infrequent, and cases of ulcerative colitis confined to the rectum were rare (1.7%). A considerable number of patients had a history of or were presently receiving treatment with anti-tumor necrosis factor medication. The degree of missing data in case mix variables was low (Table 1), below 10%, except for the SCQ, which was missing for 32% of patients.

**Table 1.** Baseline characteristics (N = 1010)

Characteristic	Missing data (%)	Data
**Demographics**		
Age, y	0	45 (33-59)
Female	0	558 (55)
Education level	11	
Lower		238 (26)
Middle		324 (36)
Higher		338 (38)
Smoking status	9.9	
Never		406 (45)
Former		351 (39)
Current		153 (17)
**Comorbidities**		
SCQ	32	1.00 (0.00-3.00)
History of TBC	10	11 (1.2)
History of hepatitis B	9.9	14 (1.5)
HIV/AIDS	10	2 (0.2)
PSC	10	17 (1.9)
**Diagnosis**		
Crohn’s disease	0	638 (63)
Ulcerative colitis	0	351 (35)
IBD unknown	0	21 (2.1)
**Montreal** **classification**		
Disease extent	0	
E1		17 (1.7)
E2		111 (11)
E3		244 (24)
Age at diagnosis	0	
A1		57 (5.6)
A2		438 (43)
A3		143 (14)
Disease location	0	
L1		152 (15)
L2		148 (15)
L3		335 (33)
L4 (isolated)		3 (0.3)
Concomitant proximal small bowel disease	0	74 (7.3)
Disease behavior	0	
B1		375 (37)
B2		157 (16)
B3		106 (10)
Perianal disease	0	162 (16)
**EIMs**		
History of dermatological EIMs	0	62 (6.1)
History of rheumatological EIMs	0	140 (14)
History of hepatobiliary EIMs	0	15 (1.5)
History of opthalmogical EIMs	0	35 (3.5)
History of other EIMs	0	1 (<0.1)
**Treatment history**		
Previous therapy with infliximab	0	343 (34)
Previous therapy with adalimumab	0	231 (23)
Previous therapy with golimumab	0	17 (1.7)
Previous therapy with vedolizumab	0	73 (7.2)
Previous therapy with ustekinumab	0	35 (3.5)
Previous therapy with tofacitinib	0	18 (1.8)
History of IBD-related surgery	0	324 (32)
**Treatment at baseline**		
Infliximab	0	348 (34)
Adalimumab	0	272 (27)
Golimumab	0	7 (0.7)
Vedolizumab	0	160 (16)
Ustekinumab	0	114 (11)
Tofacitinib	0	19 (1.9)
No biologic or new small molecule	0	90 (8.9)

Values are median (interquartile range) or n (%), unless otherwise indicated.

Abbreviations: AIDS, acquired immunodeficiency syndrome; EIM, extraintestinal manifestations; HIV, human immunodeficiency virus; IBD, inflammatory bowel disease; PSC, primary sclerosing cholangitis; SCQ, Self-Administered Comorbidity Questionnaire; TBC, tuberculosis.

### Outcomes

Outcome distribution between hospitals can be found in Supplementary Table 5 and outcome definitions can be found in Supplementary Table 1. Some variability in outcomes was observed between the hospitals in the imputed unadjusted data, for example the percentage of patients with anemia (11%-23%), patient-reported remission rates (29%-49%), long-term corticosteroid use (3.5%-9.4%), and mean number of A&E visits per patient (0.02-0.13).

### Survival and Disease Control

Outcomes and variation between hospitals for survival and disease control are shown in Table 2. There was a high percentage of missing data for the outcomes endoscopic/radiologic remission (79% and 79.4% for the first and second study periods, respectively) and biochemical remission (39.4% and 42.7%, respectively), as not all patients had an endoscopy, radiological procedure, or fecal calprotectin measurement during each 6-month period. Most patients were in clinician-reported remission (83%), but patient-reported remission rates were lower (40%). The ICCs for the hospitals for all remission outcomes were 0%, indicating that 0% of the variation between patients could be attributed to the treating hospital. However, the ICC at the patient level for most survival and disease control outcomes was above 70% (Supplementary Table 6). This means that patient characteristics that were not fully adjusted for, which include differences in disease phenotype not captured by the Montreal classification and disease course, could explain a substantial part (>70%) of the variability between patients. When looking at the highest and lowest performers, all odds ratios for the survival and disease control outcomes were very close to 1, indicating that the absolute variation in these outcomes between hospitals was very low (Table 2).

**Table 2.** Outcomes and variation for survival and disease control

Outcome	Missing data (%)	n	Overall	ICC (%)	Lowest performer (95% CI)^a^	Highest performer (95% CI)^a^
IBD-attributable mortality	0	1010	0 (0)	—	—	—
Colorectal cancer	0	1010	0 (0)	—	—	—
Anemia	9.8	1624	247 (15)	0	1.00 (1.00-1.00)	1.00 (1.00-1.00)
Endoscopic/radiologic remission	79	374	140 (37)	0	1.00 (1.00-1.00)	1.00 (1.00-1.00)
Biochemical remission	41	1057	577 (55)	0	0.99 (0.74-1.33)	1.01 (0.81-1.24)
Clinician-reported remission	0.3	1794	1483 (83)	0	1.00 (1.00-1.00)	1.00 (1.00-1.00)
Patient-reported remission (MIBDI)	21	1430	573 (40)	0	1.00 (1.00-1.00)	1.00 (1.00-1.00)

Values are n (%), unless otherwise indicated.

Abbreviations: CI, confidence interval; IBD, inflammatory bowel disease; ICC, intraclass correlation coefficient; MIBDI, Manitoba IBD index.

^a^Odds ratio [95% confidence interval].

### Healthcare Utilization and Disutility of Care

Table 3 provides insights into the variation in healthcare utilization and disutility of care. The mean number of A&E visits and admissions per 6-month period was around 0.07 and the average length of each hospital stay was 8 days. The proportion of patients visiting the A&E department or requiring admission did not vary between the hospitals (ICCs of 0% and 2%, respectively). However, differences between hospitals accounted for 7% of the variation in length of stay, meaning that 7% of the variation in length of stay between patients could be explained by the hospital at which patients were treated. In our population, 5% of the patients used corticosteroids for more than 3 months during the study period, but variation between patients could not be attributed to the treating hospital (ICC of 2%). Complications resulting from IBD-related interventions such as infusion reactions and surgical complications were rare (94 complications during 900 patient years). The variation in complications between patients was also not associated with the hospital where a patient received treatment, with an ICC of 1%. Analysis of highest and lowest performers showed some absolute variation between hospitals. An example is that patients in the lowest- and highest-performing hospitals had respectively 1.32 and 0.8 times more A&E visits compared with the mean performance. However, the wide confidence intervals around these estimates showed considerable uncertainty, and all these intervals were compatible with a situation in which the highest or lowest performers were no different than the average (Table 3).

**Table 3.** Outcomes and variation for healthcare utilization and disutility of care

Outcome	Missing data (%)	n	Overall	ICC (%)	Lowest performer (95% CI)	Highest performer (95% CI)
**Healthcare utilization**						
A&E visits	0	1010	0.07 ± 0.37	0	0.99 (0.73-1.35)^a^	1.02 (0.69-1.49)^a^
Admissions	0	1010	0.06 ± 0.32	2	0.80 (0.48-1.34)^a^	1.32 (0.73-2.40)^a^
Length of stay, d^c^	0	1010	7.99 ± 6.51	7	0.85 (0.45-1.61)^a^	1.23 (0.64-2.38)^a^
**Disutility of** c**are**^d^						
Corticosteroid use (>3 mo)	0	790	41 (5.2)	2	0.89 (0.49-1.60)^b^	1.20 (0.55-2.59)^b^
Complications	0	1010	0 (0-0)	1	0.92 (0.51-1.66)^a^	1.05 (0.72-1.52)^a^

Values are mean ± SD, n (%), or median (interquartile range), unless otherwise indicated.

Abbreviations: A&E, accident and emergency; CI, confidence interval; ICC, intraclass correlation coefficient.

^a^Relative difference.

^b^Odds ratio.

^c^For patients that had admissions.

### Symptoms, Function, and Quality of Life

Patients reported a high level of disease control based on their responses reported in the IBD-Control questionnaire with a median score of 14 (on a scale of 0 to 16), but variation between patients could not be attributed to the treating hospital, as indicated by an ICC of 0%. The sensitivity analysis on the IBD-Control-8 score with a cutoff of 13 also showed no variation on the hospital level, with an ICC of 0%. We found that approximately 1 in 8 patients had an active fistula. On average, patients reported a good quality of life (Table 4). The variation in quality-of-life outcomes between patients could not be attributed to the different hospitals, as indicated by the ICCs of 2% and lower in Table 4. Variation could be attributed to unmeasured patient characteristics, with ICCs above 40% (Supplementary Table 6). Absolute variation for all symptoms, function, and quality-of-life outcomes was very low, as shown by odds ratios near 1 for the lowest and highest performers, as well as estimated differences near 0 for the lowest and highest performers (Table 4).

**Table 4.** Outcomes and variation for symptoms, function, and quality of life

Outcome	Missing data (%)	n	Overall	ICC (%)	Lowest performer (95% CI)	Highest performer (95% CI)
**IBD-specific quality of life**						
IBD-Control-8 score	26	1333	14.0 (10.0 to 16.0)	0	0.00 (0.00 to 0.00)^a^	0.00 (0.00 to 0.00)^a^
Active fistula^d^	21	893	171 (12)	0	1.00 (1.00 to 1.00)^b^	1.00 (1.00 to 1.00)^b^
BMI, kg/m^2^	24	1373	25.6 (22.9 to 28.7)	0	1.00 (1.00 to 1.00)^c^	1.00 (1.00 to 1.00)^c^
**Generic quality of life**						
General health (PROMIS-10)	21	1425	3.00 (2.00 to 3.00)	0	1.00 (1.00 to 1.00)^c^	1.00 (1.00 to 1.00)^c^
Physical health (PROMIS-10)	38	1111	45 (40 to 51)	2	−1.11 (−2.35 to 0.14)^a^	1.18 (−0.17 to 2.53)^a^
Mental health (PROMIS-10)	30	1252	46 (41 to 51)	0	−0.18 (−1.31 to 0.95)^a^	0.18 (−0.94 to 1.30)^a^
Social health (PROMIS-10)	28	1111	3.00 (2.00 to 4.00)	2	1.00 (1.00 to 1.00)^c^	1.00 (1.00 to 1.00)^c^
Utility (EQ-5D-5L)	21	1422	0.88 (0.78 to 1.00)	0	0.00 (0.00 to 0.00)^a^	0.00 (0.00 to 0.00)^a^
Quality of life VAS (EQ-5D-5L)	21	1424	75 (64 to 85)	0	−0.15 (−1.57 to 1.26)^a^	0.25 (−1.79 to 2.29)^a^

Values are median (interquartile range) or n (%), unless otherwise indicated.

Abbreviations: BMI, body mass index; CI, confidence interval; IBD, inflammatory bowel disease; ICC, intraclass correlation coefficient; PROMIS-10, 10-item Patient-Reported Outcomes Measurement Information System Global Health Questionnaire; VAS, visual analog scale.

^a^Difference.

^b^Odds ratio.

^c^Relative difference.

^d^Only in patients with Crohn’s disease.

### Costs

Table 5 shows that the highest costs associated with IBD were costs for treatment in the hospital setting (median €8323 per 6-month period), which included inpatient care, outpatient care, diagnostics, surgery, day treatment, A&E visits, and medication. The largest contributor to these costs was costs for advanced therapies (€6611). In contrast, median primary care costs (€76), productivity costs (€0), and patient costs (€34) were much lower than costs in the hospital setting. Interestingly, the variation in costs among patients could not be attributed to the specific hospital where they received treatment but was more likely to be related to unmeasured patient characteristics (Supplementary Table 6). When looking at highest and lowest performers, most relative differences for the lowest and highest performers were close to 1, indicating little absolute difference in costs between hospitals. For patient costs, the estimates ranged between 0.87 and 1.16, indicating that estimated patient costs for the highest and lowest performers were 87% and 116% of the average costs. However, confidence intervals were wide, indicating considerable uncertainty, and were compatible, with no difference between the highest and lowest performers as compared with the average (Table 5).

**Table 5.** Costs and variation in costs

Outcome	Missing data (%)	n	Overall (€)	ICC (%)	Lowest performer (95% CI)^a^	Highest performer (95% CI)^a^
Hospital costs	0	1800	8323 (6089-11 374)	0	1.00 (0.95-1.06)	0.99 (0.92-1.07)
Costs for advanced therapies	0	1800	6611 (4884-9195)	0	1.00 (1.00-1.00)	1.00 (1.00-1.00)
Primary care costs	35	1169	76 (0-304)	0	1.00 (1.00-1.00)	1.00 (1.00-1.00)
Productivity costs	31	1245	0 (0-1619)	0	1.00 (1.00-1.00)	1.00 (1.00-1.00)
Patient costs	44	1001	34 (7-132)	1	1.16 (0.82-1.65)	0.87 (0.59-1.28)

Values are median (interquartile range), unless otherwise indicated.

Abbreviations: CI, confidence interval; ICC, intraclass correlation coefficient.

^a^Relative difference.

### Experience With Care

Patients reported positive experiences with the received care, and there was minimal variation observed between the different hospitals (Table 6). However, some areas of concern were identified, such as inadequate communication when patients had to wait for a delayed outpatient clinic visit (27% of all delayed visits). Additionally, there was variability between hospitals for inviting family members to be involved in shared decision making with regard to IBD treatment (ICC of 14%). Finally, only 75% of patients reported that adverse effects of new medication had been explained to them, suggesting that there is room for improvement. The experience measures with larger ICCs also showed larger absolute variation, in which, for example, the odds of patients being informed about a delay were 0.65 times lower for the lowest performer as compared with the average performer. However, all confidence intervals were compatible, with there being no difference between the lowest or highest performer and the mean performance.

**Table 6.** Experience with care and variation in experiences

Outcome	Missing data (%)^a^	n	Overall	ICC (%)	Lowest performer (95% CI)	Highest performer (95% CI)
Visit on time	38	475	438 (92)	0	1.00 (1.00 to 1.00)^b^	1.00 (1.00 to 1.00)^b^
Informed about delay	94	49	13 (27)	16	0.65 (0.18 to 2.35)^b^	2.53 (0.46 to 13.77)^b^
Friendly staff	58	321	309 (96)	0	0.97 (0.24 to 3.88)^b^	1.02 (0.40 to 2.62)^b^
Informed about visit	58	318	308 (97)	0	1.00 (1.00 to 1.00)^b^	1.00 (1.00 to 1.00)^b^
Prepared provider	28	549	515 (94)	1	0.91 (0.47 to 1.77)^b^	1.10 (0.56 to 2.16)^b^
Understandable answers	30	530	526 (99)	0	1.00 (1.00 to 1.00)^b^	1.00 (1.00 to 1.00)^b^
Trust in provider	25	568	542 (95)	0	1.00 (1.00 to 1.00)^b^	1.00 (1.00 to 1.00)^b^
Enough time for visit	28	551	522 (95)	0	1.00 (1.00 to 1.00)^b^	1.00 (1.00 to 1.00)^b^
Consistent information	52	362	304 (84)	1	0.94 (0.51 to 1.74)^b^	1.05 (0.62 to 1.77)^b^
Shared decision making	45	418	372 (89)	0	1.00 (1.00 to 1.00)^b^	1.00 (1.00 to 1.00)^b^
Family included in decision making	79	159	145 (91)	14	0.47 (0.13 to 1.68)^b^	2.04 (0.48 to 8.56)^b^
Informed consent	50	378	326 (86)	0	1.00 (1.00 to 1.00)^b^	1.00 (1.00 to 1.00)^b^
Adverse effects explained	58	317	233 (74)	0	1.00 (1.00 to 1.00)^b^	1.00 (1.00 to 1.00)^b^
Follow-up explained	40	455	440 (97)	0	1.00 (1.00 to 1.00)^b^	1.00 (1.00 to 1.00)^b^
Experience VAS	29	539	8.00 (8.00 to 9.00)	1	−0.12 (−0.43 to 0.19)^c^	0.10 (−0.19 to 0.39)^c^

Values are n (%) or median (interquartile range), unless otherwise indicated.

Abbreviations: CI, confidence interval; ICC, intraclass correlation coefficient; VAS, visual analog scale.

^a^Indicates missing data or if a question was not relevant for that visit.

^b^Odds ratio.

^c^Difference.

## Discussion

In this study, we assessed variation in IBD treatment for patients on advanced therapies for different outcomes, costs, and experiences in 8 hospitals to assess and improve quality of care for patients with IBD. We found that there was variation among patients in terms of outcomes, healthcare utilization, quality of life, and costs. However, variation could not be attributed to the treating hospital, but rather only to unmeasured patient characteristics. We hypothesize that these unmeasured characteristics are disease course and phenotype and could explain differences in outcomes between patients, and that these characteristics are inadequately captured by the Montreal classification, which was used to adjust for case mix differences.^41,42^ Improving the prediction of disease phenotype and disease course could enhance case mix adjustment and facilitate the implementation of personalized medicine, ultimately reducing variation among patients and improving quality of care. The variation among patients in our study could also be caused by differences in follow-up. Those patients who are more closely monitored may experience better outcomes. Additionally, the variability in patient responses to advanced therapies could be a factor, as our ability to predict the effectiveness of a specific advanced therapy for an individual patient is limited.

Previous research has emphasized the importance of reducing variation in IBD treatment to enhance appropriate and (cost-)effective care for IBD patients.^7^ Other studies have demonstrated variation between providers in structure and process measures for IBD treatment, for example the use of therapeutic drug monitoring, medication prescription, and dysplasia surveillance and management.^12-16^ Moreover, one study reported improved outcomes in patients with Crohn’s disease who were treated in academic hospitals in the United States.^17^ However, our findings revealed no variation in treatment outcomes between the 8 hospitals, of which 7 were general hospitals and 1 was an academic hospital.^11-16^

The variation found in other studies can most likely be attributed to 2 main factors. First, differences in study design: previous studies used hypothetical cases to study provider decision making, while we analyzed real-life data.^12,13,16^ Moreover, the study that found differences in outcomes between patients with Crohn’s disease in favor of academic hospitals only adjusted for a limited set of case mix variables. When comparing hospitals using outcomes, robust methods should be used to account for case mix differences and adjust for statistical uncertainty, which was not done in the previous study.^17,43,44^ We used a more extensive set for case mix adjustment and (generalized) linear mixed models to study variation attributable to hospitals more appropriately.^17^ Second, previous research focused on structure and process measures to compare quality of care between hospitals, while we emphasized outcome measures, which are more relevant to patients.^11-16^ However, outcome measures may be less sensitive than process measures, necessitating larger sample sizes, and might be subject to residual confounding or measurement differences between hospitals.^45^ In our study, there might have been residual confounding despite the extensive case mix adjustment, or sample size issues, although over 1000 patients were included in the study.

A previous systematic review in other diseases found that the proportion of explained variation in performance measures, including outcomes, costs, experience, and process measures, was often quite low at the hospital level (<20%).^40^ We found even less variation attributable to the hospitals (<7%), and most attributable to unmeasured patient characteristics, indicating that differences between patients are more likely to explain differences in outcomes than differences between hospitals. Treatment variation might also have been lower than expected due to the collaborative efforts of the hospitals in the IBD BeterKeten initiative.

To gain more insight in the variation between hospitals, we also assessed the effect estimates of the lowest and highest performers. These analyses were performed because even though the percentage of attributable variation was low; if total variation was high, the variation might still be clinically relevant.^40^ However, none of these effect estimates were statistically significant different from the mean performance. As the variation in outcomes could not be attributed to the treating hospitals, and none of the highest or lowest performers could be determined as better or worse than average, it seems that outcome variation alone is not informative enough to be used in quality improvement initiatives for IBD care in hospitals. Although outcome variation might not be sufficient, insight in the outcomes and trends over time is still important to ensure adequate quality of care, for example by tracking them to assess the effect of quality-of-care programs.^46^

Concerning outcomes of care, our findings can be compared to a study that assessed part of the ICHOM standard set in a group of NHS hospitals.^8^ Patients in our study indicated a high level of disease control, comparable to the NHS study, and reported a satisfactory quality of life. However, while long-term corticosteroid use in our study is lower than in the NHS study (5% vs 8%), this was still relatively high, as the goal of IBD treatment is to achieve steroid-free remission. As our population only consisted of IBD patients on an advanced therapy, the difference might be due to the fact that the NHS study looked at all IBD patients irrespective of treatment, in which 37% of patient used advanced therapies. The difference in proportion of patients on an advanced therapy and with complex disease might have influenced the need for corticosteroids. Moreover, we found an average of 0.07 A&E visits and 0.06 hospital admissions per patient per 6-month period of the study, which is approximately comparable to the NHS study in which admission rates and A&E visits were 18% and 14% over a 12-month period, respectively. An exact comparison was not possible due to differences in the data collection and analysis methods. Long-term corticosteroid use, hospital admissions, and A&E visits should be prevented when possible. Tracking these outcomes in a quality improvement program could be used to assess the effect of interventions that aim to reduce steroid use, admissions, and A&E visits.^46^

Interestingly, patient-reported remission rates (40%) were substantially lower than clinician-reported rates (83%). Combined with the finding that there was little data on objective measures of remission such as fecal calprotectin and endoscopy in clinical practice, it raises the question whether the STRIDE-II (Selecting Therapeutic Targets in Inflammatory Bowel Disease II) recommendations are always followed when treating IBD patients.^47^ Overall, patients experiences with care were positive, although there is room for improvement in communication about delays in outpatient visits and side effects, as well as in the shared decision-making process.

An important but not surprising finding in our study is the high costs incurred for treating IBD in a hospital setting. In this population treated with advanced therapies, the main cost driver was the use of biologics and new small molecules. There is some evidence that maintenance therapy with biologics is not cost-effective.^7,48^ As costs of IBD treatment are rapidly increasing worldwide,^3^ new treatment strategies that focus on effective use of expensive medication are urgently needed. It is crucial for future studies to adopt a more value-based approach to IBD care by considering both outcomes and costs of treatment.^19^

Based on our results and previous studies, the best way to improve quality of IBD care seems to be the use of structure and process measures to identify care deficits using existing consensus-based quality-of-care standards. Structure measures relate to the context in which care is delivered, while process measures are the steps that providers take while treating patients. An example of a structure measure for quality of IBD care is the presence of a specialized IBD nurse at the IBD unit, and a process measure for IBD care could be following the STRIDE-II criteria during follow-up.^47,49^ Hospitals can compare their outcomes with the quality-of-care standards and address deficits. This should be combined with measuring outcomes longitudinally, as they are what matter most to patients and can be used to provide feedback on quality improvement initiatives. Continually aiming to improve the structure and process of care while assessing the effect of interventions on outcomes can lead to a learning health system. These systems, as exemplified by the ImproveCareNow network and IBD Qorus, have shown to lead to improvements in the care of IBD patients by improving remission rates and reducing corticosteroid use, hospital admissions, and A&E visits.^46,50-52^

The main strengths of this study are the prospective design, comprehensive range of outcome measures, large sample size (including almost 25% of the entire eligible population), and use of robust statistical methods and case mix adjustment to assess variation between hospitals in IBD care. Moreover, we used the ICHOM Standard Set for patient-centered outcomes of IBD care to assess differences between hospitals. This standard set was developed based on literature, patient input, and specialist consensus. Our study is the first to comprehensively study the differences in outcomes, costs, and experiences of IBD care among hospitals. Because of the robust methodology and use of a standardized outcomes framework, it can serve as a guide for future national and international comparisons on the quality of IBD care. However, there are limitations to consider. First, the inclusion of only patients on biologics and new small molecules limits the generalizability of our results to the general IBD population. Patients that are not on advanced therapies but could have benefited from them are not included in our study population, and there might be variation in when different hospitals start advanced therapies, leading to differences in treatment outcomes for this population. This inclusion criterion was chosen as the IBD Value project focused on improving care for this complex and refractory group of patients with IBD.^23^ Second, we included approximately 25% of all eligible patients. While the sample size is quite large, there might be differences between patients who did and did not participate in the study, leading to possible selection bias. Third, despite extensive case mix adjustment, residual confounding may obscure true variation between hospitals, as seen in a recent study in stroke patients.^53^ Fourth, only patients treated in 8 hospitals in the Netherlands were eligible for inclusion. As gastroenterologists working in these hospitals often received specialty training from the same academic institution and sometimes collaborate in multidisciplinary meetings for complex cases, treatment variation might be lower than when random hospitals would have been sampled. Fifth, monitoring of patients was not standardized, as we were interested in variation in a real-world setting. However, differences in how often patients were monitored might have led to bias and missing data. Missing data were relatively low for case mix variables, and these were multiply imputed to reduce bias when estimating the variation between hospitals. However, the proportion of missing data was considerably higher for endoscopic and radiologic remission and biochemical remission because frequency of monitoring depended on local protocols. These estimates are likely still biased after imputation. As the degree and direction of bias in these outcomes cannot be quantified, the degree of variation between hospitals in this outcome cannot be reliably interpreted. Last, the follow-up period of 1 year might be too short to show variation in outcomes due to differences in when and how therapy is escalated or switched, as some advanced therapies need a longer time to induce benefit.

## Conclusions

Our study shows that differences in outcomes, costs, and patient experiences in IBD care for patients on advanced therapies are not linked to the treating hospital but are instead associated with variations among patients. Because relying solely on outcome measures is insufficient for evaluating and comparing the quality of IBD care across hospitals, future initiatives for enhancing quality should look at differences in structure and process measures between hospitals. Additionally, there should be a focus on patient-level interventions to personalize care and minimize variation among patients.

## Supplementary Material

izae095_suppl_Supplementary_Material

## Data Availability

Data are available upon reasonable request. The dataset generated in this study will be available on reasonable request.
