# Supplementary material for: Variation Between Hospitals in Outcomes and Costs of IBD Care: Results From the IBD Value Study
Source: Inflamm Bowel Dis. 2024 Apr 26;31(2):332–43. doi: 10.1093/ibd/izae095 (PMC11808576; doi:10.1093/ibd/izae095)
Supplement: izae095_suppl_Supplementary_Material [file izae095_suppl_supplementary_material.pdf]

SUPPLEMENTARY METHODS

Case mix

We removed infection with HIV and 'other' extraintestinal manifestations from the ICHOM case-mix set in the analyses as these were very rare and led to convergence issues in the regression models. Patient-reported diagnosis of PSC was removed from the analyses as this was highly collinear with extraintestinal hepatobiliary manifestations.

Imputation procedure

The few patients that were lost to follow-up were considered to not have died due to IBD and not have developed colorectal cancer. Other missing data was imputed using Multivariate Imputation by Chained Equations with the mice package in R.[1] We used an imputation, then deletion approach for the outcome variables, so only case mix variables were imputed. This approach was chosen because imputation of outcomes in mixed model settings can lead to bias. However, imputing case mix variables in this setting leads to an increased sample size, improved power and lower bias as compared to complete case analysis.[2,3]

All outcomes and case mix variables from the analyses were added in the imputation model. Treating hospital was added as a fixed effect to reduce the biasing effect of shrinkage during the imputation procedure, while a random effect was used for clustering on patient level.[3] Medication history, medication at baseline, and surgical history were added as auxiliary variables to improve imputation. Costs were rescaled using a fourth root and number of admissions, cumulative length of stay and BMI with a square root to improve convergence of imputation models. All missing data were imputed using predictive mean matching.

A total of 25 datasets was imputed using 50 iterations. Convergence of the imputed values was assessed using plots of the trace lines. Density and bar plots of the observed and imputed values were used to assess whether imputed values were plausible. The intraclass correlation coefficient (ICC) was calculated by taking the mean of ICC over the imputed datasets in accordance with Rubin's rules.[3]

Statistical analysis

All outcomes, costs and experiences with care were analysed using (generalised) linear mixed models. Most outcomes were analysed at the 6 and 12 month mark, and these models contained patient level and hospital level random effects to account for clustering. Some outcomes were

analysed over the entire baseline period: cumulative length of stay, number of admissions, number of A&E visits and long-term corticosteroid use. These models only contained a hospital level random effect. All random effects were assumed to follow a normal distribution. See Supplementary Table 3 for details on the specification of each model. Analyses were performed in R version 4.2.0 and RStudio version 2022.02.2.485.[4,5] Models were fit using the glmmTMB package (version 1.1.7.900) and the ICC was calculated using the performance package (version 0.10.3).[6,7] Appropriateness of the distributions and link functions used was checked with the DHARMa package (version 0.4.6).[8]

SUPPLEMENTARY TABLES

Outcome	Source	Definition
**Survival and Disease Control**		
IBD-attributable mortality	Chart review	Death due to IBD or IBD-related complication
Colorectal cancer	Chart review	Incident colorectal cancer
Anaemia	Medical record	Presence of anaemia[9], lowest haemoglobin in 6 month period<br>Men: Hb < 8.07 mmol/L<br>Women: Hb < 7.45 mmol/L
Endoscopic/radiologic remission	Chart review	No inflammation on endoscopy or radiology
Biochemical remission	Medical record	FCP below cut-off, highest FCP in 6 month period<br>CD: < 100<br>UC/IBD-U: < 250
Clinical remission	Chart review	In remission according to treating provider
Patient-reported remission	Patient-reported	A few days of complaints or less in the last 6 months according to the MIBDI[10]
**Healthcare Utilisation**		
A&E visits	Medical record	Number of A&E visits for IBD-related specialties
Admissions	Medical record	Number of hospital admissions for IBD-related specialties, excluding day treatment
Length of stay	Medical record	Cumulative length of stay for IBD-related specialties
**Disutility of Care**		
Long-term steroid use	Medical record	>3 months corticosteroid use in 12 month period
Complications	Chart review	Number of complications of IBD treatment
**Symptoms, Function, and Quality of Life**		
Patient-reported disease control	Patient-reported	IBD-Control-8 score[11,12]
Fistulae symptoms	Patient-reported	Self-reported active fistula
BMI	Patient-reported	Weight divided by height in meters squared
**Generic Quality of Life**		
General health	Patient-reported	General health score on the PROMIS-10[13]
Physical health	Patient-reported	Physical health score on the PROMIS-10[13]
Mental health	Patient-reported	Mental health score on the PROMIS-10[13]
Social health	Patient-reported	Social health score on the PROMIS-10[13]
Utility	Patient-reported	Utility according to the EQ-5D-5L[14] and Dutch tariffs[15]
Quality of life VAS	Patient-reported	Quality of life according to the EQ-5D-5L VAS[14]

**Costs**		
Hospital costs	Medical record	Hospital costs consisting of admissions, surgery, day treatment, outpatient clinic visits, diagnostics, medication (including biologics) and A&E visits for IBD-related specialties valued with Dutch reference prices[16-18] adjusted for inflation to 2022[19]
Biological or new small molecule costs	Medical record	Costs for biologics or new small molecules used to treat IBD valued with Dutch reference prices[18] adjusted for inflation to 2022[19]
Primary care costs	Patient-reported	Primary care costs consisting of visits to the general practitioner, social worker, physiotherapist, dietician, psychologist, psychiatrist, and occupational physician, and costs for ambulance rides and home care, measured with the iMCQ[20] and valued with Dutch reference prices[16-18] adjusted for inflation to 2022[19]
Productivity costs	Patient-reported	Productivity costs consisting of absenteeism, presenteeism and loss of unpaid work measured with the iPCQ[21] and valued with Dutch reference prices[16] adjusted for inflation to 2022[22]
Patient costs	Patient-reported	Patient costs consisting of travel, over-the-counter drugs, informal care and other IBD-related costs, valued with Dutch reference prices[16] adjusted for inflation to 2022[19]
**Patient experience**		
Visit on time	Patient-reported	If the outpatient consultation was on time (PEM)[23]
Informed about delay	Patient-reported	If the patient was informed about the delay (PEM)[23]
Friendly staff	Patient-reported	If the staff at reception was friendly (PEM)[23]
Informed about visit	Patient-reported	If the patient was informed about what was going to happen during the visit (PEM)[23]
Prepared provider	Patient-reported	If the provider read the information in the medical record (PEM)[23]
Understandable answers	Patient-reported	If the answers to questions were understandable (PEM)[23]
Trust in provider	Patient-reported	If the patient trusted the provider (PEM)[23]
Enough time for visit	Patient-reported	If there was enough time for the consultation (PEM)[23]
Consistent information	Patient-reported	If information from different providers was consistent (PEM)[23]
Shared decision-making	Patient-reported	If the patient was involved in treatment decisions (PEM)[23]
Family included in decision-making	Patient-reported	If the patient's family was involved in treatment decisions (PEM)[23]
Informed consent	Patient-reported	If pros and cons of treatment were explained (PEM)[23]
Adverse effects explained	Patient-reported	If possible side effects of new medication were explained (PEM)[23]
Follow-up explained	Patient-reported	If the follow-up schedule was explained (PEM)[23]
Experience VAS	Patient-reported	Patient experience with care on a VAS (PEM)[23]

Supplementary Table 1. Outcomes and their respective source and definition. Adapted from van Linschoten et al.[24]

Abbreviations: A&E: Accident & emergency; CD: Crohn's disease; Hb: Hemoglobin; IBD: Inflammatory bowel disease; IBD-U: IBD-unknown; MIBDI: Manitoba IBD Index; PEM: Patient-experience monitor; PROMIS-10: Patient-reported outcomes measurement information system global health questionnaire; iMTA: Institute of Medical Technology Assessment; iPCQ: iMTA Productivity Cost Questionnaire; iMCQ: iMTA Medical Consumption Questionnaire; UC: Ulcerative colitis; VAS: Visual analogue scale

**Case mix variable**	**Source**	**Definition**
Period	-	Time point in study (6 or 12 months)
Age	Chart review	Age at inclusion
Sex	Chart review	Sex at birth
Education level	Patient-reported	Low, middle or high according to UNESCO[25]
Smoking status	Patient-reported	Current, ex or never smoker
Diagnosis	Chart review	CD, UC, or IBD-U
Montreal classification	Chart review	For UC and IBD-U: extent<br>For CD: age, localisation and behaviour
Comorbidities	Patient-reported	Measured with the SCQ[26] with the exclusion of anaemia/blood disease as this is an outcome in the analyses
Tuberculosis	Patient-reported	Prior or current infection with tuberculosis
Hepatitis B	Patient-reported	Prior or current infection with hepatitis B
Extraintestinal skin manifestations	Chart review	History of pyoderma gangrenosum, erythema nodosum, cutaneous CD, or hidradenitis suppurativa
Extraintestinal joint manifestations	Chart review	History of ankylosing spondylitis or IBD-related joint inflammation
Extraintestinal eye manifestations	Chart review	History of uveitis, scleritis or episcleritis
Extraintestinal hepatobiliary manifestations	Chart review	History of primary sclerosing cholangitis

Supplementary Table 2. Case mix variables and their respective source and definition.
Abbreviations: CD: Crohn's disease; IBD: Inflammatory bowel disease; IBD-U: IBD-unknown; SCQ: Self-administered comorbidity questionnaire; UC: Ulcerative colitis

**Outcome**	**Distribution (link function)**	**Notes**
**Survival and Disease Control**		
IBD-attributable mortality	-	Not enough outcomes (n=0) to fit model
Colorectal cancer	-	Not enough outcomes (n=0) to fit model
Anaemia	Binomial (Logit)	TBC and hepatitis B removed from case-mix variables due to convergence issues
Endoscopic/radiologic remission	Binomial (Logit)	
Biochemical remission	Binomial (Logit)	
Clinical remission	Binomial (Logit)	TBC and hepatitis B removed from case-mix variables due to convergence issues
Patient-reported remission	Binomial (Logit)	TBC and extraintestinal hepatobiliary manifestations removed from case-mix variables due to convergence issues
**Healthcare Utilisation**		
A&E visits	Negative Binomial (Log)	Analysed over 12 months with an offset to account for different follow-up periods
Admissions	Negative Binomial (Log)	Analysed over 12 months with an offset to account for different follow-up periods
Length of stay	Zero-inflated Gamma (Log)	Analysed over 12 months with an offset to account for different follow-up periods<br>Zero-inflation model: only intercept
**Disutility of Care**		
Long-term steroid use	Binomial (Logit)	Analysed over 12 months for patients with 12 months follow-up<br>TBC and extraintestinal hepatobiliary manifestations removed from case-mix variables due to convergence issues
Complications	Negative Binomial (Log)	Analysed over 12 months with an offset to account for different follow-up periods<br>Extraintestinal hepatobiliary manifestations removed from case-mix variables due to convergence issues
**Symptoms, Function, and Quality of Life**		
Patient-reported disease control	Gaussian (Identity)	
Fistulae symptoms	Binomial (Logit)	Only in patients with Crohn’s disease<br>Extraintestinal hepatobiliary manifestations removed from case-mix variables due to convergence issues
BMI	Gaussian (Log)	
**Generic Quality of Life**		
General health	Generalised Poisson (Log)	
Physical health	Gaussian (Identity)	
Mental health	Gaussian (Identity)	

Social health	Generalised Poisson (Log)	
Utility	Gaussian (Identity)	
Quality of life VAS	Gaussian (Identity)	
**Costs**		
Hospital costs	Gamma (Log)	
Biological costs	Zero-inflated Gamma (Log)	Zero-inflation model: SCQ, age and education level
Primary care costs	Zero-inflated Gamma (Log)	Zero-inflation model: SCQ, age and education level
Productivity costs	Zero-inflated Gamma (Log)	Zero-inflation model: SCQ, age and education level
Patient costs	Zero-inflated Gamma (Log)	Zero-inflation model: SCQ, age and education level
**Patient experience**		
Visit on time	Binomial (Logit)	
Informed about delay	Binomial (Logit)	
Friendly staff	Binomial (Logit)	
Informed about visit	Binomial (Logit)	
Prepared provider	Binomial (Logit)	
Understandable answers	Binomial (Logit)	
Trust in provider	Binomial (Logit)	
Enough time for visit	Binomial (Logit)	
Consistent information	Binomial (Logit)	
Shared decision-making	Binomial (Logit)	
Family included in decision-making	Binomial (Logit)	
Informed consent	Binomial (Logit)	
Adverse effects explained	Binomial (Logit)	
Follow-up explained	Binomial (Logit)	
Experience VAS	Gaussian (Identity)	

Supplementary Table 3. Outcomes and their respective model definition
Abbreviations: A&E: Accident & emergency; SCQ: Self-administered comorbidity questionnaire; VAS: Visual analogue scale

Characteristic	Hospital 1<br>N = 112	Hospital 2<br>N = 137	Hospital 3<br>N = 154	Hospital 4<br>N = 148	Hospital 5<br>N = 140	Hospital 6<br>N = 68	Hospital 7<br>N = 141	Hospital 8<br>N = 110
**Demographics**[a]								
Age	46 (36, 57)	45 (33, 60)	50 (33, 60)	48 (36, 61)	42 (34, 57)	44 (31, 58)	37 (29, 51)	46 (34, 62)
Female	72 (64%)	67 (49%)	81 (53%)	84 (57%)	69 (49%)	32 (47%)	93 (66%)	60 (55%)
Education level								
Lower	22 (23%)	34 (27%)	32 (23%)	39 (30%)	31 (22%)	20 (34%)	32 (29%)	28 (29%)
Middle	37 (39%)	46 (36%)	47 (33%)	46 (35%)	53 (38%)	16 (27%)	44 (40%)	35 (37%)
Higher	37 (39%)	47 (37%)	63 (44%)	45 (35%)	56 (40%)	23 (39%)	35 (32%)	32 (34%)
Smoking status								
Never	45 (45%)	51 (40%)	58 (41%)	57 (44%)	66 (47%)	34 (57%)	52 (46%)	43 (44%)
Ex	36 (36%)	50 (39%)	67 (47%)	51 (39%)	48 (34%)	17 (28%)	38 (34%)	44 (45%)
Current	18 (18%)	27 (21%)	18 (13%)	22 (17%)	26 (19%)	9 (15%)	23 (20%)	10 (10%)
**Comorbidities**[a]								
SCQ	1.0 (0.0, 2.0)	0.0 (0.0, 2.0)	1.0 (0.0, 3.0)	1.0 (0.0, 2.0)	2.0 (0.0, 4.3)	2.0 (0.0, 3.0)	0.0 (0.0, 2.0)	1.0 (0.0, 3.0)
History of TBC	2 (2.0%)	2 (1.6%)	1 (0.7%)	3 (2.3%)	2 (1.4%)	0 (0%)	1 (0.9%)	0 (0%)
History of Hepatitis B	1 (1.0%)	0 (0%)	2 (1.4%)	3 (2.3%)	2 (1.4%)	1 (1.7%)	3 (2.7%)	2 (2.1%)
HIV/AIDS	0 (0%)	0 (0%)	0 (0%)	0 (0%)	0 (0%)	1 (1.7%)	0 (0%)	1 (1.0%)
PSC	2 (2.0%)	1 (0.8%)	2 (1.4%)	0 (0%)	10 (7.2%)	0 (0%)	2 (1.8%)	0 (0%)
**Diagnosis**[a]								
Crohn's disease	61 (54%)	78 (57%)	102 (66%)	92 (62%)	105 (75%)	45 (66%)	92 (65%)	63 (57%)
Ulcerative colitis	49 (44%)	56 (41%)	48 (31%)	55 (37%)	27 (19%)	23 (34%)	49 (35%)	44 (40%)
IBD-Unknown	2 (1.8%)	3 (2.2%)	4 (2.6%)	1 (0.7%)	8 (5.7%)	0 (0%)	0 (0%)	3 (2.7%)
**Montreal Classification**[a]								
Disease extent								
E1	5 (4.5%)	2 (1.5%)	3 (1.9%)	3 (2.0%)	1 (0.7%)	1 (1.5%)	2 (1.4%)	0 (0%)
E2	14 (13%)	15 (11%)	12 (7.8%)	20 (14%)	10 (7.1%)	4 (5.9%)	20 (14%)	16 (15%)
E3	32 (29%)	42 (31%)	37 (24%)	33 (22%)	24 (17%)	18 (26%)	27 (19%)	31 (28%)
Age at diagnosis								
A1	3 (2.7%)	4 (2.9%)	6 (3.9%)	8 (5.4%)	22 (16%)	4 (5.9%)	4 (2.8%)	6 (5.5%)
A2	43 (38%)	53 (39%)	65 (42%)	57 (39%)	69 (49%)	28 (41%)	80 (57%)	43 (39%)
A3	15 (13%)	21 (15%)	31 (20%)	27 (18%)	14 (10%)	13 (19%)	8 (5.7%)	14 (13%)

Disease location								
L1	16 (14%)	18 (13%)	29 (19%)	22 (15%)	26 (19%)	10 (15%)	13 (9.2%)	18 (16%)
L2	16 (14%)	26 (19%)	20 (13%)	24 (16%)	24 (17%)	9 (13%)	20 (14%)	9 (8.2%)
L3	29 (26%)	34 (25%)	52 (34%)	45 (30%)	55 (39%)	25 (37%)	59 (42%)	36 (33%)
L4 (isolated upper disease)	0 (0%)	0 (0%)	1 (0.6%)	1 (0.7%)	0 (0%)	1 (1.5%)	0 (0%)	0 (0%)
L4 (concomitant upper disease)	2 (1.8%)	6 (4.4%)	11 (7.1%)	13 (8.8%)	20 (14%)	6 (8.8%)	12 (8.5%)	4 (3.6%)
Disease behaviour								
B1	41 (37%)	44 (32%)	56 (36%)	44 (30%)	50 (36%)	26 (38%)	70 (50%)	44 (40%)
B2	8 (7.1%)	22 (16%)	25 (16%)	25 (17%)	38 (27%)	9 (13%)	16 (11%)	14 (13%)
B3	12 (11%)	12 (8.8%)	21 (14%)	23 (16%)	17 (12%)	10 (15%)	6 (4.3%)	5 (4.5%)
Perianal disease	8 (7.1%)	20 (15%)	26 (17%)	24 (16%)	37 (26%)	11 (16%)	18 (13%)	18 (16%)
**Extra-intestinal manifestations**[a]								
History of dermatological EIMs	9 (8.0%)	5 (3.6%)	9 (5.8%)	9 (6.1%)	7 (5.0%)	3 (4.4%)	10 (7.1%)	10 (9.1%)
History of rheumatological EIMs	14 (13%)	5 (3.6%)	11 (7.1%)	21 (14%)	22 (16%)	15 (22%)	33 (23%)	19 (17%)
History of hepatobiliary EIMs	3 (2.7%)	0 (0%)	0 (0%)	1 (0.7%)	8 (5.7%)	0 (0%)	2 (1.4%)	1 (0.9%)
History of opthalmogical EIMs	2 (1.8%)	1 (0.7%)	6 (3.9%)	6 (4.1%)	5 (3.6%)	3 (4.4%)	5 (3.5%)	7 (6.4%)
History of other EIMs	0 (0%)	0 (0%)	0 (0%)	0 (0%)	1 (0.7%)	0 (0%)	0 (0%)	0 (0%)
**Treatment history**[a]								
Previous therapy with infliximab	22 (20%)	47 (34%)	35 (23%)	47 (32%)	80 (57%)	24 (35%)	55 (39%)	33 (30%)
Previous therapy with adalimumab	20 (18%)	27 (20%)	23 (15%)	40 (27%)	51 (36%)	14 (21%)	27 (19%)	29 (26%)
Previous therapy with golimumab	2 (1.8%)	2 (1.5%)	5 (3.2%)	2 (1.4%)	5 (3.6%)	0 (0%)	1 (0.7%)	0 (0%)
Previous therapy with vedolizumab	5 (4.5%)	10 (7.3%)	13 (8.4%)	14 (9.5%)	22 (16%)	3 (4.4%)	2 (1.4%)	4 (3.6%)
Previous therapy with ustekinumab	0 (0%)	3 (2.2%)	5 (3.2%)	2 (1.4%)	12 (8.6%)	5 (7.4%)	4 (2.8%)	4 (3.6%)
Previous therapy with tofacitinib	0 (0%)	1 (0.7%)	8 (5.2%)	2 (1.4%)	3 (2.1%)	3 (4.4%)	0 (0%)	1 (0.9%)
History of IBD-related surgery	25 (22%)	32 (23%)	59 (38%)	53 (36%)	73 (52%)	19 (28%)	36 (26%)	27 (25%)
**Treatment at baseline**[a]								
Infliximab	36 (32%)	54 (39%)	48 (31%)	59 (40%)	32 (23%)	22 (32%)	57 (40%)	40 (36%)
Adalimumab	34 (30%)	31 (23%)	41 (27%)	39 (26%)	47 (34%)	20 (29%)	24 (17%)	36 (33%)
Golimumab	2 (1.8%)	0 (0%)	1 (0.6%)	0 (0%)	2 (1.4%)	2 (2.9%)	0 (0%)	0 (0%)
Vedolizumab	18 (16%)	22 (16%)	24 (16%)	16 (11%)	27 (19%)	5 (7.4%)	28 (20%)	20 (18%)
Ustekinumab	8 (7.1%)	23 (17%)	18 (12%)	18 (12%)	19 (14%)	8 (12%)	14 (9.9%)	6 (5.5%)
Tofacitinib	0 (0%)	0 (0%)	5 (3.2%)	4 (2.7%)	5 (3.6%)	1 (1.5%)	0 (0%)	4 (3.6%)

No biologic	14 (13%)	7 (5.1%)	17 (11%)	12 (8.1%)	8 (5.7%)	10 (15%)	18 (13%)	4 (3.6%)

Supplementary Table 4. Patient characteristics per hospital

[a] Median (IQR); n (%)

Abbreviations: EIM: Extra-intestinal manifestations; HIV/AIDS: Human immunodeficiency virus/acquired immunodeficiency syndrome; IBD: Inflammatory bowel disease; IQR: Interquartile range; N = number of patients; PSC: Primary sclerosing cholangitis; SCQ: Self-administered comorbidity questionnaire; TBC: Tuberculosis

Outcome	Hospital 1<br>N = 259	Hospital 2<br>N = 279	Hospital 3<br>N = 245	Hospital 4<br>N = 261	Hospital 5<br>N = 206	Hospital 6<br>N = 252	Hospital 7<br>N = 189	Hospital 8<br>N = 109
**Survival and Disease Control[a]**								
IBD-attributable mortality	0 (0%)	0 (0%)	0 (0%)	0 (0%)	0 (0%)	0 (0%)	0 (0%)	0 (0%)
Colorectal cancer	0 (0%)	0 (0%)	0 (0%)	0 (0%)	0 (0%)	0 (0%)	0 (0%)	0 (0%)
Anaemia	52 (23%)	26 (11%)	36 (15%)	38 (16%)	25 (14%)	39 (16%)	20 (12%)	11 (11%)
Endoscopic/radiologic remission	36 (43%)	18 (36%)	20 (43%)	18 (35%)	15 (35%)	14 (38%)	10 (32%)	9 (28%)
Biochemical remission	84 (48%)	105 (56%)	46 (54%)	77 (49%)	49 (62%)	75 (59%)	94 (62%)	47 (51%)
Clinician-reported remission	214 (83%)	247 (89%)	212 (87%)	219 (84%)	166 (82%)	190 (76%)	157 (83%)	78 (72%)
Patient-reported remission (MIBDI)	82 (36%)	105 (45%)	59 (36%)	78 (38%)	46 (29%)	99 (47%)	73 (49%)	31 (37%)
**Healthcare Utilisation[b]**								
A&E visits	0.08 (0.31)	0.05 (0.26)	0.13 (0.61)	0.10 (0.44)	0.02 (0.15)	0.06 (0.30)	0.06 (0.29)	0.07 (0.33)
Admissions	0.05 (0.23)	0.04 (0.20)	0.11 (0.47)	0.11 (0.46)	0.05 (0.28)	0.05 (0.25)	0.04 (0.24)	0.06 (0.28)
Length of stay[c]	6.64 (3.11)	9.22 (13.14)	8.69 (6.65)	8.95 (5.79)	6.50 (7.13)	7.60 (4.20)	8.40 (5.32)	5.83 (3.19)
**Disutility of Care[a]**								
Corticosteroid use (>3 m)[d]	4 (3.5%)	10 (8.0%)	5 (4.7%)	11 (9.4%)	3 (3.1%)	3 (2.6%)	3 (4.0%)	2 (4.8%)
Complications	0 (0, 0)	0 (0, 0)	0 (0, 0)	0 (0, 0)	0 (0, 0)	0 (0, 0)	0 (0, 0)	0 (0, 0)
**Symptoms, Function, and Quality of Life[a]**								
IBD-Control-8 score	12.0 (9.0, 15.0)	14.0 (11.0, 16.0)	14.0 (10.0, 15.0)	14.0 (11.0, 16.0)	14.0 (11.0, 16.0)	14.0 (11.0, 15.0)	14.0 (11.0, 16.0)	13.0 (9.0, 15.0)
Active fistula	36 (16%)	26 (11%)	22 (13%)	23 (11%)	25 (16%)	20 (9.7%)	8 (5.4%)	11 (13%)
BMI	24.8 (22.1, 27.7)	26.0 (24.0, 29.3)	26.0 (23.6, 28.2)	24.8 (22.7, 27.6)	26.3 (23.5, 29.6)	25.5 (22.7, 28.5)	26.5 (23.7, 29.4)	26.8 (22.6, 29.3)
**Generic Quality of Life[a]**								
General health (PROMIS)	2.00 (2.00, 3.00)	3.00 (2.00, 3.00)	3.00 (2.00, 3.00)	3.00 (2.00, 3.00)	3.00 (2.00, 3.00)	3.00 (2.00, 3.00)	3.00 (2.00, 3.00)	3.00 (2.00, 3.00)
Physical health (PROMIS)	42 (35, 48)	45 (40, 51)	45 (40, 48)	48 (40, 54)	45 (40, 48)	45 (40, 51)	46 (40, 54)	42 (40, 48)
Mental health (PROMIS)	46 (39, 51)	46 (41, 48)	46 (41, 51)	46 (41, 53)	46 (41, 52)	46 (41, 51)	46 (39, 51)	44 (39, 48)
Social health (PROMIS)	3.00 (2.00, 4.00)	3.00 (2.00, 3.50)	3.00 (2.00, 4.00)	3.00 (3.00, 4.00)	3.00 (2.50, 4.00)	3.00 (2.00, 4.00)	3.00 (2.00, 4.00)	3.00 (2.00, 3.00)
Utility (EQ-5D-5L)	0.85 (0.77, 1.00)	0.88 (0.77, 1.00)	0.86 (0.79, 0.91)	0.89 (0.77, 1.00)	0.86 (0.79, 0.91)	0.86 (0.75, 1.00)	0.89 (0.78, 1.00)	0.85 (0.75, 0.96)
Quality of life VAS (EQ-5D-5L)	75 (62, 81)	75 (65, 85)	75 (64, 85)	79 (66, 90)	75 (61, 81)	75 (65, 85)	77 (65, 87)	73 (65, 89)

**Costs[a]**								
Hospital costs	€8,330 (€6,300, €11,738)	€8,156 (€5,986, €10,278)	€8,617 (€6,375, €11,476)	€8,657 (€6,348, €13,014)	€8,494 (€6,124, €12,018)	€8,363 (€5,916, €11,095)	€8,042 (€5,207, €10,359)	€8,176 (€6,032, €11,411)
Biological costs	€6,896 (€4,598, €9,195)	€6,512 (€4,884, €8,517)	€6,644 (€4,884, €9,195)	€6,867 (€4,884, €9,768)	€6,896 (€4,884, €9,744)	€6,512 (€4,623, €8,328)	€6,512 (€3,868, €8,328)	€6,124 (€4,835, €9,195)
Primary care costs	€114 (€38, €394)	€76 (€0, €259)	€76 (€0, €266)	€76 (€0, €266)	€76 (€0, €266)	€113 (€0, €332)	€38 (€0, €252)	€76 (€19, €190)
Productivity costs	€0 (€0, €2,214)	€0 (€0, €2,341)	€0 (€0, €2,163)	€0 (€0, €784)	€0 (€0, €962)	€0 (€0, €994)	€0 (€0, €1,483)	€0 (€0, €1,391)
Patient costs	€95 (€29, €378)	€45 (€8, €133)	€38 (€11, €81)	€18 (€0, €70)	€28 (€8, €132)	€18 (€0, €77)	€21 (€0, €105)	€25 (€4, €70)
**Patient Experience[a]**								
Visit on time	43 (86%)	80 (94%)	57 (90%)	83 (93%)	50 (94%)	67 (94%)	38 (86%)	20 (100%)
Informed about delay	5 (63%)	1 (13%)	1 (10%)	1 (13%)	0 (0%)	3 (60%)	2 (33%)	0 (0%)
Friendly staff	26 (96%)	39 (93%)	45 (98%)	69 (93%)	40 (100%)	45 (98%)	31 (100%)	14 (93%)
Informed about visit	31 (100%)	53 (96%)	41 (98%)	53 (96%)	40 (93%)	44 (98%)	29 (100%)	17 (94%)
Prepared provider	51 (91%)	90 (91%)	64 (90%)	95 (96%)	56 (93%)	78 (98%)	56 (98%)	25 (93%)
Understandable answers	55 (98%)	95 (99%)	64 (100%)	92 (99%)	62 (100%)	73 (100%)	56 (98%)	29 (100%)
Trust in provider	56 (93%)	96 (93%)	72 (99%)	95 (95%)	59 (95%)	77 (95%)	59 (97%)	28 (100%)
Enough time for visit	55 (95%)	97 (95%)	70 (99%)	91 (94%)	55 (95%)	73 (92%)	57 (95%)	24 (92%)
Consistent information	31 (79%)	51 (86%)	41 (87%)	62 (89%)	29 (74%)	41 (77%)	35 (92%)	14 (82%)
Shared decision-making	34 (87%)	67 (89%)	51 (93%)	62 (84%)	41 (89%)	57 (88%)	44 (96%)	16 (89%)
Family included in decision-making	8 (89%)	21 (91%)	18 (95%)	25 (81%)	25 (96%)	35 (100%)	9 (75%)	4 (100%)
Informed consent	32 (86%)	54 (83%)	47 (89%)	52 (83%)	40 (91%)	53 (88%)	29 (88%)	19 (83%)
Adverse effects explained	15 (65%)	38 (72%)	29 (71%)	44 (75%)	31 (82%)	41 (76%)	24 (75%)	11 (65%)
Follow-up explained	38 (97%)	84 (97%)	50 (98%)	77 (99%)	55 (98%)	64 (96%)	50 (93%)	22 (96%)
Experience VAS	8.00 (8.00, 9.00)	8.00 (8.00, 9.00)	8.00 (8.00, 9.00)	8.00 (8.00, 9.00)	9.00 (8.00, 9.00)	8.00 (8.00, 9.00)	8.00 (8.00, 9.00)	8.00 (8.00, 9.00)

Supplementary Table 5. Outcomes, costs and experience with care stratified per hospital.

[a] Median (IQR); n (%)

[b] Mean

[c] For patients that had admissions

[d] Over the entire 12 month study period for patients that had 12 months of follow-up

Abbreviations: A&E: Accidents and emergency; BMI: Body mass index; IBD: Inflammatory bowel disease; IQR: Interquartile range; MIBDI: Manitoba IBD Index; N = number of observations; PROMIS: Patient-reported outcomes measurement information system; VAS: Visual analogue scale

Outcome	ICC Hospital	ICC Patient
**Survival and Disease Control**		
IBD-attributable mortality	-	-
Colorectal cancer	-	-
Anaemia	0%	98%
Endoscopic/radiologic remission	0%	7%
Biochemical remission	0%	35%
Clinician-reported remission	0%	96%
Patient-reported remission (MIBDI)	0%	73%
**Healthcare Utilisation**		
A&E visits	0%	-
Admissions	2%	-
Length of stay[c]	7%	-
**Disutility of Care**		
Corticosteroid use (>3 m)[d]	2%	-
Complications	1%	-
**Symptoms, Function, and Quality of Life**		
IBD-Control-8 score	0%	53%
IBD-Control-8 score (dichotomised)	0%	45%
Active fistula	0%	99%
BMI	0%	96%
**Generic Quality of Life**		
General health (PROMIS)	0%	70%
Physical health (PROMIS)	2%	74%
Mental health (PROMIS)	0%	73%
Social health (PROMIS)	2%	74%
Utility (EQ-5D-5L)	0%	64%
Quality of life VAS (EQ-5D-5L)	0%	58%
**Costs**		
Hospital costs	0%	45%
Biologic costs	0%	61%
Primary care costs	0%	66%
Productivity costs	0%	51%
Patient costs	1%	78%

Supplementary Table 6. Intraclass correlation coefficients of all outcome and cost analyses

Abbreviations: -: no random effect at this level; BMI: Body mass index; IBD: Inflammatory bowel disease; ICC: Intraclass correlation coefficient; MIBDI: Manitoba IBD Index; PROMIS: Patient-reported outcomes measurement information system; VAS: Visual analogue scale

SUPPLEMENTARY REFERENCES

1. van Buuren S, Groothuis-Oudshoorn K. Mice: Multivariate imputation by chained equations in r. *Journal of Statistical Software* 2011;**45**:1-67.
2. von Hippel PT. Regression with missing ys: An improved strategy for analyzing multiply imputed data. *Sociol Methodol* 2007;**37**:83-117.
3. Van Linschoten RC, Amini M, Van Leeuwen N, Eijkenaar F, Den Hartog SJ, Nederkoorn PJ, Hofmeijer J, Emmer BJ, Postma AA, Van Zwam W. Handling missing values in the analysis of between-hospital differences in ordinal and dichotomous outcomes: A simulation study. *BMJ Quality & Safety* 2023.
4. R Core Team. R: A language and environment for statistical computing. Vienna, Austria: R Foundation for Statistical Computing, 2023.
5. RStudio Team. Rstudio: Integrated development for r. Boston, MA: RStudio, PBC., 2023.
6. Borooks ME, Kristensen K, Benthem KJ, Magnusson A, Berg CW, Nielsen A, Skaug HJ, Maechler M, Bolker BM. Glmmtmb balances speed and flexibility among packages for zero-inflated generalized linear mixed modeling. *The R Journal* 2017;**9**:378-400.
7. Lüdecke D, Ben -Schachar MS, Patil I, Waggoner P, Makowski D. Performance: An r package for assessment, comparison and testing of statistical models. *Journal of Open Source Software* 2021;**6**:3139.
8. Hartig F. Dharma: Residual diagnostics for hierarchical (multi-level / mixed) regression models. . 2022.
9. World Health Organization. Haemoglobin concentrations for the diagnosis of anaemia and assessment of severity. World Health Organization, 2011.
10. Clara I, Lix LM, Walker JR, Graff LA, Miller N, Rogala L, Rawsthorne P, Bernstein CN. The manitoba IBD index: Evidence for a new and simple indicator of IBD activity. *Am J Gastroenterol* 2009;**104**:1754-63.
11. de Jong ME, Taal E, Thomas PWA, Römkens TEH, Jansen JM, West RL, Slotman E, Hoentjen F, Russel M. Cross-cultural translation and validation of the IBD-control questionnaire in the netherlands: A patient-reported outcome measure in inflammatory bowel disease. *Scand J Gastroenterol* 2021;**56**:155-61.
12. Bodger K, Ormerod C, Shackcloth D, Harrison M, Collaborative IBDC. Development and validation of a rapid, generic measure of disease control from the patient's perspective: The IBD-control questionnaire. *Gut* 2014;**63**:1092-102.
13. Hays RD, Bjorner JB, Revicki DA, Spritzer KL, Cella D. Development of physical and mental health summary scores from the patient-reported outcomes measurement information system (promis) global items. *Qual Life Res* 2009;**18**:873-80.
14. Herdman M, Gudex C, Lloyd A, Janssen MF, Kind P, Parkin D, Bonsel G, Badia X. Development and preliminary testing of the new five-level version of eq-5d (eq-5d-5l). *Qual Life Res* 2011;**20**:1727-36.
15. Versteegh MM, Vermeulen KM, Evers SMAA, de Wit GA, Prenger R, Stolk EA. Dutch tariff for the five-level version of eq-5d. *Value Health* 2016;**19**:343-52.
16. Hakkaart-van Roijen L, Van der Linden N, Bouwmans C, Kanters T, SS T. Kostenhandleiding. Methodologie van kostenonderzoek en referentieprijzen voor economische evaluaties in de gezondheidszorg.: Zorginstituut Nederland, 2015.
17. CZ. Tarieven medisch specialistische zorg per 1 januari 2022. https://www.cz.nl/-/media/2022/voorwaarden/gemiddeld-ongewogen-gecontracteerde-tarieven-msz.pdf?revid=c01bd1eb-8f58-484c-a1ad-17b384e1e0dd Accessed 04-10-2022, 2021.
18. National Health Care Institute. Medicijnkosten. https://www.medicijnkosten.nl/ Accessed 18-06-2019, 2019.
19. Centraal Bureau voor Statistiek. Consumentenprijzen; prijsindex 2015=100. https://opendata.cbs.nl/statline#/CBS/nl/dataset/83131NED/table?ts=1649249699079 Accessed 20-04-2022, 2022.

20. iMTA Productivity and Health Research Group. Handleiding imta medical cost questionnaire (imcq). www.imta.nl, 2018.
21. Bouwmans C, Krol M, Severens H, Koopmanschap M, Brouwer W, Hakkaart-van Roijen L. The imta productivity cost questionnaire: A standardized instrument for measuring and valuing health-related productivity losses. *Value Health* 2015;**18**:753-8.
22. Centraal Bureau voor Statistiek. Prijs van arbeid; index 2015=100; nationale rekeningen. https://opendata.cbs.nl/#/CBS/nl/dataset/84183NED/table Accessed 20-04-2022, 2022.
23. Bastemeijer CM, Boosman H, Zandbelt L, Timman R, de Boer D, Hazelzet JA. Patient experience monitor (pem): The development of new short-form picker experience questionnaires for hospital patients with a wide range of literacy levels. *Patient Related Outcome Measures* 2020;**Volume 11**:221-30.
24. van Linschoten RCA, van Leeuwen N, Nieboer D, Birnie E, Scherpenzeel M, Verweij KE, de Jonge V, Hazelzet JA, van der Woude CJ, West RL, van Noord D. Value-based care pathway for inflammatory bowel disease: A protocol for the multicentre longitudinal non-randomised parallel cluster IBD value study with baseline period. *BMJ Open* 2022;**12**.
25. UNESCO Institute for Statistics. International standard classification of education: Isced 2011. UNESCO Institute for Statistics Montreal, 2012.
26. Sangha O, Stucki G, Liang MH, Fossel AH, Katz JN. The self-administered comorbidity questionnaire: A new method to assess comorbidity for clinical and health services research. *Arthritis Rheum* 2003;**49**:156-63.
